# Detection and Characterisation of *Lactobacillus* spp. in the Bovine Uterus and Their Influence on Bovine Endometrial Epithelial Cells *In Vitro*

**DOI:** 10.1371/journal.pone.0119793

**Published:** 2015-03-24

**Authors:** Martina A. Gärtner, Angelika Bondzio, Nicole Braun, Markus Jung, Ralf Einspanier, Christoph Gabler

**Affiliations:** 1 Institute of Veterinary Biochemistry, Department of Veterinary Medicine, Freie Universität Berlin, Berlin, Germany; 2 Institute for the Reproduction of Farm Animals Schönow, Bernau, Germany; Rutgers University, UNITED STATES

## Abstract

Bacterial infections and inflammation of the uterus are common in dairy cattle after parturition. In particular, pathogenic bacteria that cause endometritis have been the focus of research in cattle reproduction in the last ten years. The aim of the present study was to identify commensal lactobacilli in the bovine uterus and to examine their influence on the synthesis of pro-inflammatory factors in bovine endometrial epithelial cells *in vitro*. *Lactobacillus* species were isolated from healthy bovine uteri and further characterised. Bovine endometrial epithelial cells in the second passage (n = 5 animals) were co-cultured with the autochthonous isolates *L*. *buchneri*, *L*. *ruminis* and *L*. *amylovorus* as well as with a commercially available *L*. *vaginalis* in different multiplicities of infection (MOI = 1, 5 and 10, respectively). Endometrial epithelial cells cultured without bacteria served as controls. At distinct points in time (2, 4 and 6 h) total RNA was extracted from co-cultured epithelial cells and subjected to reverse transcription quantitative PCR of pro-inflammatory factors. Furthermore, the release of such factors by co-cultured epithelial cells was measured by ELISA or EIA after 24 and 48 h. *L*. *ruminis* and *L*. *amylovorus* induced increased interleukin (IL) *IL1A*, *IL6*, *IL8* and prostaglandin-endoperoxide synthase 2 mRNA levels and the release of IL8 and prostaglandin F_2α_ in endometrial epithelial cells compared with control cells. In contrast, *L*. *buchneri* did not significantly influence the expression and release of these factors. Toll-like receptors 2 and 6 transcripts were found unchanged in co-cultured and untreated epithelial cells *in vitro*. However, endometrial epithelial cells of each animal showed individual differences in the response to bacterial load. These results suggest that *Lactobacillus* species are present in the bovine uterus, revealing immunomodulatory properties.

## Introduction

Uterine bacterial invasion is common in dairy cattle after parturition. A wide range of different bacteria were isolated from samples collected from the uterus. Many studies have focused on pathogens such as *Trueperella pyogenes*, *Escherichia coli*, *Fusobacterium necrophorum*, *Prevotella* spp., *Staphylococcus* spp. and *Streptococcus* spp. [1–4]. However, commensal bacteria such as the genus *Lactobacillus* were detected in the uterus of cows, but not further investigated. Few studies have mentioned the culture of lactobacilli from uterine samples of cows [5,6], but their role in the uterine environment has not yet been elucidated. In the bovine vagina, the presence of lactobacilli has been shown during the growth of healthy heifers [7] and during the luteal phase of the oestrous cycle [8]. Lactobacilli are predominant in the human vaginal tract [9], where they play a role in maintaining the vaginal ecosystem in a healthy status by producing hydrogen peroxide, acetic and lactic acid and antibacterial molecules for a first defence against bacteria [10–12].

Bacteria that ascend into the uterus are recognised by endometrial epithelial cells via Toll-like receptors (TLRs). These proteins are able to detect pathogen-associated molecular patterns (PAMPs) such as lipopolysaccharide (LPS) from Gram-negative bacteria and lipoteichoic acid from Gram-positive bacteria [13]. This recognition of invading pathogens is the first step of immune response of the endometrium followed by producing pro-inflammatory cytokines such as interleukins and chemokines to attract polymorphonuclear neutrophils (PMNs) to the uterine lumen for bacterial clearance [14]. Molecules involved in the inflammatory processes in the endometrium include the cytokines interleukin 1 alpha (IL1A), IL6 and IL8 [15] and prostaglandins whose production is under the regulation of prostaglandin-endoperoxide synthase 2 (PTGS2) [16]. If the inflammation of the endometrium caused by bacterial infection is excessive or persistent, it results in the development of uterine diseases [15]. It has been shown that genital diseases have negative consequences for fertility by increasing the calving-to-conception interval and reducing conception rates [14,17,18].

Several studies have demonstrated that cows with genital diseases showed enhanced expression of interleukins and *PTGS2*. Cows with subclinical and clinical endometritis revealed increased mRNA expression of *IL1A* [19] and *IL8* [20] in endometrial cytobrush samples compared with healthy cows. Furthermore, cows with inflamed endometrium showed enhanced *IL6*, *IL8* and *PTGS2* expression around two weeks postpartum (pp) compared with healthy endometrium [21,22]. The increased *IL6* and *IL8* expression in endometrial biopsies of endometritic cows compared with healthy cows supports these findings [23]. The results of these studies indicate that these interleukins as well as *PTGS2* are involved in the immune response in inflammatory endometrial diseases.

The hypothesis of increased expression of interleukins and *PTGS2* in cows with endometritis caused by a bacterial infection is supported by *in vitro* studies of endometrial cells co-cultured with pathogenic bacteria. Higher *IL8* expression was observed after treatment of endometrial epithelial cells with LPS purified from endometrial pathogenic *E*. *coli* [24]. Additionally, *PTGS2* was more highly expressed in bovine endometrial epithelial cells after stimulation with *E*. *coli* or LPS compared with control cells [16]. Not only pathogenic bacteria, but also commensal bacteria have been shown to upregulate pro-inflammatory factors. *L*. *rhamnosus* and *L*. *reuteri* increased *IL1A* expression in human vaginal epithelial cells [25], and IL6 as well as IL8 production by peripheral blood mononuclear cells was induced by *L*. *fermentum* and *L*. *salivarius* [26].

In contrast to the human or bovine vagina, lactobacilli have not been in the focus of studies concerning the bovine uterus so far and their role in the endometrial microbiota is unclear. Therefore, the aim of this study was to isolate and characterise lactobacilli from the bovine uterus and to examine their influence on the expression and production of pro-inflammatory factors in bovine endometrial epithelial cells *in vitro*.

## Materials and Methods

### Isolation and culturing of *Lactobacillus* species

Cows were kept on two commercial dairy farms in freestall facilities in the state of Brandenburg (Germany) in accordance with the guidelines of the National Animal Welfare Legislation, and the local animal welfare officer (Berlin, Germany) approved the study. These bacterial samples from the dairy farm cows were taken by professional veterinarians as part of routine diagnostics. For this reason, no ethics committee approval is necessary because such an approach did not lead to any additional pain or discomfort-inducing manipulations for the cows during the entire study. The procedure of taking cytobrush samples from the uterus is similar to artificial insemination. Therefore, no sedation or local anaesthesia was applied. None of these cows had to be sacrificed because of the sampling.

Non-pregnant animals without signs of genital diseases were chosen for this study. Bacteriological samples of the endometrium were collected from 11 cows 40 to 90 days pp. One sample from each cow was taken using the cytobrush technique as described previously with slight modifications [27]. A sterile brush (Cytobrush Plus GT, Medscand Medical, Malmö, Sweden) was covered with a metallic catheter. To protect the cytobrush from vaginal and external contaminations, the catheter was enveloped by a sanitary plastic sleeve. The catheter was placed via the cervix into the uterine body and the sleeve was retracted followed by gently pushing the cytobrush into the uterine lumen. A bacterial sample was taken by rolling the cytobrush in contact with the uterine wall. The samples were stored in MRS broth (Sigma-Aldrich, Steinheim, Germany) and transported to the laboratory at room temperature. Samples were cultured aerobically on Rogosa SL agar and LBS agar (both Sigma-Aldrich) at 37°C for up to 72 h to detect lactobacilli. Bacteria of single colonies were picked and subsequently grown in MRS broth followed by streaking on agar plates. This was repeated three times. Bacteria were plated on blood agar (Merck, Darmstadt, Germany) to ensure the purity of the bacterial strains. All isolates were stored at -80°C in 15% glycerol until further experiments.

### Characterisation of isolated bacteria

Bacterial species were identified by the characteristics of Gram stain, colony morphology, catalase reaction, nitrate reduction and indole production. For a first screening, a PCR with *Lactobacillus*-specific primers [28] based on the ribosomal ribonucleic acid (rRNA) gene was performed. Briefly, the forward primer was based on the flanking terminal sequence of the 16S rRNA gene (5‘-CTT GTA CAC ACC GCC CGT CA-3’). The sequence of the 16S to 23S ribosomal RNA intergenic spacer region of *L*. *acidophilus* starting at position 70 was used as reverse primer (5’-CTC AAA ACT AAA CAA AGT TTC-3’). Both primers were synthesised by Eurofins MWG (Ebersberg, Germany). A single colony of each isolate plated on MRS agar was picked and cultured in MRS broth for 24 h. The grown bacterial suspension was diluted 10x with TE buffer (10 mM Tris-HCl, 1 mM EDTA, pH 8.0) and stored at 4°C until use. PCR amplifications were performed in a thermocycler with a 25 μl total volume containing 0.4 μM of each primer, 0.4 mM each dNTP, 3 mM MgCl_2_, 1x Immobuffer, 0.5 U Immolase (all Fermentas, St. Leon-Roth, Germany) and 5 μl of the diluted bacterial suspension mentioned above. Cycling conditions were as follows: 10 min at 95°C, followed by 40 cycles of 1 min at 95°C, 1 min at 55°C and 1 min at 72°C, and a 3 min final extension step at 72°C. PCR amplicons were analysed by 2% agarose gel electrophoresis containing ethidium bromide. An approximately 250 bp long PCR product was obtained.

Bacteria with amplicons obtained in this PCR approach were used for characterisation of extended products by sequencing. For that, DNA was extracted from 24 h bacterial cultures in MRS broth by using a QIAamp DNA Mini Kit (Qiagen, Hilden, Germany) following the manufacturer’s instructions. Isolated DNA was quantified spectrophotometrically at a wavelength of 260 nm and subjected to a PCR using phylogenetic 16S rDNA primers [29]. Briefly, the reaction mixture mentioned above was used except for 1.5 mM MgCl_2_ and 200 ng of genomic bacterial DNA (primer for: 5’-AGA GTT TGA TCC TGG CTC AG-3’, primer rev: 5’-AAG GAG GTG ATC CAG CC-3’; synthesised by Eurofins MWG). Amplification of DNA fragments was performed as follows: 10 min at 95°C, 35 cycles of 95°C for 2 min, 42°C for 30 s and 72°C for 4 min, followed by a final elongation step at 72°C for 20 min. The PCR amplicons were subjected to 1% agarose gel electrophoresis containing ethidium bromide and extracted from the gel using an Invisorb Spin DNA Extraction Kit (Stratec, Berlin, Germany) according to the manufacturer’s instructions. The forward and reverse sequences of the PCR products were obtained (GATC Biotech, Konstanz, Germany) followed by comparing the resulting sequences with the NCBI database to obtain the specific bacterial strain sequence information.

### Isolation and culture of endometrial epithelial cells

All media, antibiotics and serum used for cell culture were supplied by Biochrom (Berlin, Germany). Isolation of bovine endometrial epithelial cells was performed as described previously [30] with slight modifications. Briefly, bovine uteri were collected from healthy, non-pregnant cows at a local slaughterhouse (Schlachtbetrieb GmbH Perleberg, Perleberg, Germany) approximately 15 min after death. For that, permission from the slaughterhouse to collect the uteri was obtained. The cows were slaughtered by the staff of the slaughterhouse according to standard procedures. Uteri were transported on ice to the laboratory. Pieces of the endometrium were dissected, minced very finely and placed into 25 ml of Hanks’ Balanced Salt Solution (HBSS) containing 150 U/ml collagenase (Sigma-Aldrich), 150 U/ml hyaluronidase (Sigma-Aldrich), 200 U/ml penicillin and 200 μg/ml streptomycin for incubation at 37°C with mild agitation for 2 h. After one washing and trituration step, cells were suspended in Dulbecco’s modified Eagle’s medium (DMEM)/Ham’s F-12 medium containing 10% fetal bovine serum (FBS) superior, 55 μg/ml gentamicin and 1.4 μg/ml amphotericin B. The cells were plated in 25 cm^2^ culture flasks (Corning, Corning, USA) for 18 h, which allowed selective attachment of stromal cells. An epithelial cell culture was obtained by removing and reseeding the suspension after this time [24]. Immunocytochemistry against pan-keratins was performed for proofing of cell type and purity of epithelial cells as described previously [31]. Cells were incubated at 37°C and 5% CO_2_ in a humidified atmosphere. Epithelial cell populations of the second passage with not more than 5% stromal cell contamination were used for further experiments.

### Co-culture of endometrial epithelial cells with different *Lactobacillus* species

Bovine endometrial epithelial cells were cultured in the first passage until reaching confluency. Then they were seeded in 24-well plates (Greiner Bio-One, Frickenhausen, Germany) in a density of 8 x 10^4^ cells in 500 μl medium for viability assay, enzyme-linked immunosorbent assay (ELISA) or enzyme immunoassay (EIA) as well as in 6-well plates (Greiner Bio-One) with 3 x 10^5^ cells in 4 ml medium for mRNA expression analysis. After reaching confluency in the second passage, the medium was removed and cells were washed twice with Dulbecco’s phosphate-buffered saline (PBS; PAA, Cölbe, Germany).

Isolated lactobacilli and *L*. *vaginalis* (purchased from Deutsche Sammlung von Mikroorganismen und Zellkulturen, Braunschweig, Germany) were prepared for co-culturing with endometrial epithelial cells by growing in MRS broth at 37°C for 48 h after storing in glycerol at -80°C. Bacteria were harvested by centrifugation for 10 min at 3800 g, washed once with PBS, resuspended in PBS and stored in aliquots at -80°C. The number of colony-forming units (CFU)/ml in aliquots was determined by plate counting on MRS agar after thawing. For co-culturing with epithelial cells, aliquots of lactobacilli were thawed and diluted in DMEM/Ham’s F-12 medium without antibiotics in different multiplicities of infection (MOI = 1, 5 and 10, respectively). Epithelial cells in wells with medium without bacteria served as controls. To detect the influence on the viability of the cells, lactobacilli were co-cultured with epithelial cells for up to 96 h. mRNA expression analysis was performed after up to 6 h of co-culturing. Up to 48 h after the beginning of co-culture, the release of pro-inflammatory factors by endometrial epithelial cells was determined by ELISA or EIA.

### Viability assay

After 72 and 96 h of co-culture, the medium was aspirated and epithelial cells were washed twice with PBS. Cell viability was determined by trypan blue exclusion test [32]. Dead cells were stained blue with a 1:1 mixture of 0.5% (w/v) trypan blue (Serva, Heidelberg, Germany) and PBS. In addition, cell nuclei were stained with fluorescent DNA dyes [33]. A mixture of ethidium bromide (10 mg/ml; Sigma Aldrich) and acridine orange (5 mg/ml; Sigma Aldrich) in PBS was added to the washed epithelial cells and immediately observed using a fluorescence microscope (Axiovert 35, Carl Zeiss, Oberkochen, Germany) with excitation by blue light (wavelength 450–490 nm). Five different optical fields were considered and pictures were taken. Epithelial cells cultured with the same medium without bacteria served as controls. Experiments were conducted using epithelial cells isolated from five different animals.

### Extraction of total RNA and reverse transcription after co-culturing

mRNA expression of pro-inflammatory factors was analysed in co-cultured endometrial epithelial cells. For this purpose, the medium was removed after 2, 4 and 6 h of co-culture, and epithelial cells were washed twice with PBS and lysed with Lysis buffer TR (Stratec). Control cells were also lysed at 0 h. The lysates were stored at -80°C until use. Total RNA was extracted from the lysates by an InviMag Universal RNA Kit (Stratec) using the KingFisher Flex (Thermo Scientific, Langenselbold, Germany) according to the manufacturer’s instructions. The yield of total RNA was estimated spectrophotometrically at 260 nm and isolated RNA was stored at -80°C. RNA quality and integrity was verified using the Agilent 2100 Bioanalyzer (RNA 6000 Nano Chip, Agilent, Waldbronn, Germany).

cDNA was synthesised out of 1 μg of total RNA using 2.5 μM random hexamers, 0.66 mM dNTPs, 1x RT buffer and 200 U RevertAid reverse transcriptase (all Fermentas) in a total volume of 60 μl [34]. Treatment with DNAse I (Fermentas) was performed before reverse transcription to remove possible genomic DNA contaminations. The generated cDNA was stored at -20°C until use. Reactions omitting the reverse transcriptase served as negative controls to monitor the absence of any genomic DNA or contaminations.

### Quantitative PCR

Quantitative PCR (qPCR) in the presence of SYBR Green I was performed using the Rotor Gene 3000 (Corbett Research, Mortlake, Australia) as described in detail [34] following the MIQE guidelines [35]. The 10 μl reaction mixture consisted of 1 μl cDNA, 1x SensiMix Low-ROX (Bioline, Luckenwalde, Germany) and 0.4 μM of each primer (primer pairs are given in Table 1; synthesised by Eurofins MWG). The following cycling conditions were performed: 10 min at 95°C, 45 cycles of 15 s at 95°C, 20 s at the indicated annealing temperature (Table 1) and 30 s at 72°C. Melting point analysis of the amplified products confirmed specific amplification. For mRNA quantification, a dilution series of PCR products with known concentrations generated in a conventional PCR was amplified simultaneously with the samples as a standard. Quantities of specific mRNA were calculated using the standard curves and Rotor Gene 6.1 software (Corbett Research). Amplicons were commercially sequenced (GATC Biotech) and showed a 100% homology to known bovine sequences. For normalisation of mRNA expression, beta actin (*ACTB*), succinate dehydrogenase complex, subunit A (*SDHA*) and glyceraldehyde 3-phosphate dehydrogenase (*GAPDH*) (Table 1) were used as reference genes. These selected reference genes were stably expressed.

**Table 1:** Selected gene transcripts, primer sequences and annealing temperatures used for qPCR with resulting amplicon length.

Gene	Primer sequence	Reference	Fragment size (bp)	Annealing temperature
*IL1A*	for 5'-TCA TCC ACC AGG AAT GCA TC-3'	[19]	300 bp	59°C
	rev 5'-AGC CAT GCT TTT CCC AGA AG-3'			
*IL6*	for 5'-TCC AGA ACG AGT ATG AGG-3'	[68]	236 bp	56°C
	rev 5'-CAT CCG AAT AGC TCT CAG-3'			
*IL8*	for 5'-CGA TGC CAA TGC ATA AAA AC-3'	[20]	153 bp	56°C
	rev 5'-CTT TTC CTT GGG GTT TAG GC-3'			
*PTGS2*	for 5'-CTC TTC CTC CTG TGC CTG AT-3'	[34]	359 bp	60°C
	rev 5'-CTG AGT ATC TTT GAC TGT GGG AG-3'			
*TLR2*	for 5'-GTA CCC ATG ATG GAA TTG GC-3'	NM_174197	446 bp	60°C
	rev 5'-TGG CCA CTG ACA AGT TTC AG-3'			
*TLR6*	for 5'-GGA AAG CTA CAA GGG AAC CC-3'	NM_001001159	276 bp	60°C
	rev 5'-ACC CAG GCA GAG TCA TGT TC-3'			
*ACTB*	for 5'-CGG TGC CCA TCT ATG AGG-3'	AY141970	266 bp	58°C
	rev 5'-GAT GGT GAT GAC CTG CCC-3'			
*SDHA*	for 5'-GGG AGG ACT TCA AGG AGA GG-3'	BT030722	219 bp	60°C
	rev 5'-CTC CTC AGT AGG AGC GGA TG-3'			
*GAPDH*	for 5'-CCC AGA AGA CTG TGG ATG G-3'	U85042	306 bp	62°C
	rev 5'-AGT CGC AGG AGA CAA CCT G-3'			

### ELISA and EIA

Supernatants of the co-culture of endometrial epithelial cells with isolated lactobacilli and *L*. *vaginalis* were analysed for concentrations of IL6 and IL8 by ELISA, as well as of prostaglandin F_2α_ (PGF_2α_) and prostaglandin E_2_ (PGE_2_) by EIA. Concentrations were measured after 24 and 48 h of co-culture using commercially available kits according to the manufacturer’s instructions [Bovine IL-6 Screening Set (Thermo Scientific); Human CXCL8/IL-8 DuoSet (R&D Systems, Wiesbaden, Germany); Prostaglandin F_2α_ EIA Kit (Cayman Chemical, Ann Arbor, USA); Prostaglandin E_2_ EIA Kit (Cayman Chemical)]. Dilutions of the samples within the detection range were used for each ELISA or EIA. Cross-reaction of the antibody pairs of Human CXCL8/IL-8 DuoSet to bovine IL8 has previously been shown [36]. Cell culture supernatants were centrifuged twice (400 g and 16,200 g for 5 min, respectively) after harvesting to remove epithelial cells and bacteria. Supernatants were stored at -80°C until measurement. Each experiment was conducted in duplicate using endometrial epithelial cells isolated from three different animals. Concentrations of pro-inflammatory factors in cell culture supernatants were estimated using a microtitre reader (iMark Bio Rad, Bio-Rad Laboratories, Munich, Germany) by comparing with standard curves.

### Statistical analysis

For relative quantification of the mRNA expression of the investigated factors, absolute quantities of each gene were divided through the corresponding normalisation factor calculated with the expression of the reference genes *ACTB*, *SDHA* and *GAPDH* by geNorm [37]. Normalised data were analysed by the Wilcoxon signed-rank test by comparing each MOI to the control at the same point in time. The normalised expression in controls and treatments at each point in time was calculated relative to the expression in control cells at 0 h that was set equal to one. Bar charts were generated using the relative values with bars representing the means ± SEM (n = 5).

All values of pro-inflammatory factors in cell culture supernatants were scaled relative to the concentration in control cell supernatants after 24 h that was defined as one. Bar charts represent the means ± SEM (n = 3).

All statistical calculations were performed by using SPSS version 20 (SPSS, Chicago, USA). Values of P < 0.5 were considered to be significant.

## Results

### Characterisation of isolated bacteria

Uterine cytobrush samples collected from 11 healthy lactating cows were analysed for the presence of lactobacilli. Gram-positive, catalase-negative, indole-negative and nitrate reduction-negative micro-organisms were identified as lactobacilli [38]. Samples from two animals were culture-negative. All culture-positive samples showed slight bacterial growth (<10 colonies). Only catalase-positive colonies grew on the agar plates of three samples. These bacteria were not further identified because this was not the focus of this study. A *Lactobacillus*-specific PCR revealed nine isolates belonging to the *Lactobacillus* group with a product size of approximately 250 bp (data not shown). Sequencing with phylogenetic 16S rDNA primers identified these isolates as *L*. *buchneri*, *L*. *ruminis*, *L*. *amylovorus*, *L*. *plantarum* and *L*. *similis*. Three isolates were determined as the closest relatives to *Weissella paramesenteroides* and one to *Pediococcus pentosaceus*. The identity of the isolates in comparison to the NCBI database is listed in Table 2.

**Table 2:** Qualitative characterisation of isolated uterine lactic acid bacteria.

Animal #	Identified Species	Type Strain	% Identity to Type Strain	Accession #
1	*Lactobacillus plantarum*	BS16	99	JX968493.1
	*Pediococcus pentosaceus*	4	99	HG328247.1
	*Weissella paramesenteroides*	FMA204	99	HQ721255.1
2	*Lactobacillus similis*	LZLJ10–3	99	JQ043373.1
	*Weissella paramesenteroides*	CTSPL5	99	EU855224.1
3	*Lactobacillus buchneri*	NRRL B-30929	99	CP002652.1
4	*Lactobacillus ruminis*	ATCC27782	100	CP003032.1
5	*Weissella paramesenteroides*	TR7.1.15	99	HQ009793.1
6	*Lactobacillus amylovorus*	GRL1112	99	NR_075048.1

### Viability assay

The trypan blue exclusion test was performed 96 h after the beginning of co-culture staining dead cells blue. Almost the same quantity of dead cells was observed in the co-culture of endometrial epithelial cells with *L*. *buchneri*, *L*. *ruminis*, *L*. *amylovorus* and *L*. *vaginalis* compared with control (>95% viable cells; S1 Fig.). Cell nuclei were stained with the fluorescent dyes ethidium bromide and acridine orange 72 h after the start of co-culturing, with dead cells appearing red and living cells green. The amount of dead cells in co-culture with *L*. *buchneri*, *L*. *ruminis*, *L*. *amylovorus* and *L*. *vaginalis* in all MOI was similar to control cells up to 72 h (up to 5%; S2 Fig.). Culturing of supernatants on MRS agar showed that lactobacilli were still viable after 24 and 48 h of co-culture (data not shown). It was observed that half-confluent epithelial cells reached confluence in the presence of *L*. *vaginalis*, *L*. *buchneri*, *L*. *ruminis* and *L*. *amylovorus* (data not shown).

### *IL1A* mRNA expression in endometrial epithelial cells after co-culturing with different *Lactobacillus* species

**Fig 1:**
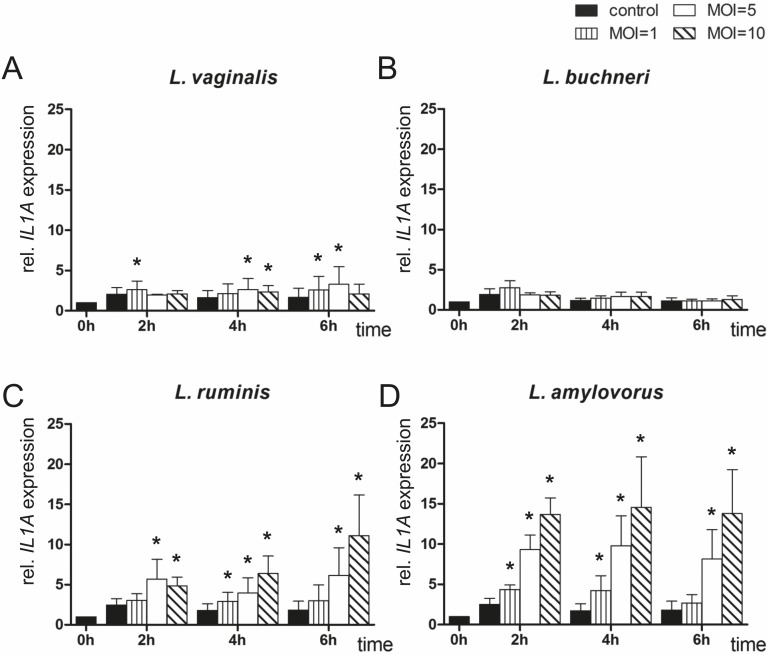
Expression analysis of *IL1A* by means of RT-qPCR. *IL1A* mRNA expression was determined in bovine endometrial epithelial cells after co-culture with (A) *L*. *vaginalis*; (B) *L*. *buchneri*; (C) *L*. *ruminis*; and (D) *L*. *amylovorus* in MOI 1, 5 and 10 for up to 6 h (n = 5 animals). All expression values were calculated relative to the individual expression of *ACTB*, *GAPDH* and *SDHA* as internal control. Normalised data were scaled relative to the expression in control cells at time 0 h, which was defined as one. Bars represent means ± SEM; * = P < 0.05.

*IL1A* mRNA expression was measured in bovine endometrial epithelial cells co-cultured with different *Lactobacillus* species for up to 6 h (Fig. 1). The presence of *L*. *vaginalis* only slightly, but significantly, influenced *IL1A* expression in co-cultured cells compared with controls (max. twofold, Fig. 1 A). In contrast, *L*. *buchneri* caused no significant difference in mRNA expression of *IL1A* independently of the time of co-culture (Fig. 1 B). *IL1A* expression increased over the time of co-culturing, with *L*. *ruminis* reaching the highest level (∼sixfold) after 6 h in MOI 10 compared with control cells (Fig. 1 C). mRNA expression of *IL1A* was on a similar level in endometrial epithelial cells co-cultured with *L*. *amylovorus* at each point in time, but increased from MOI 1 to 10 (up to eightfold) compared with cells cultured without bacteria (Fig. 1 D).

### *IL6* mRNA expression in endometrial epithelial cells after co-culturing with different *Lactobacillus* species

**Fig 2:**
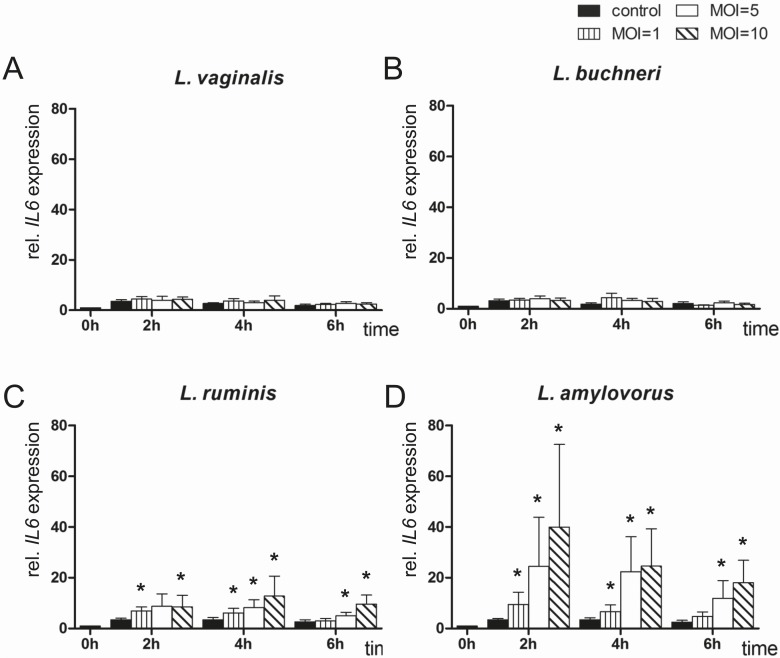
Expression analysis of *IL6* by means of RT-qPCR. *IL6* mRNA expression was determined in bovine endometrial epithelial cells after co-culture with (A) *L*. *vaginalis*; (B) *L*. *buchneri*; (C) *L*. *ruminis*; and (D) *L*. *amylovorus* in MOI 1, 5 and 10 for up to 6 h (n = 5 animals). All expression values were calculated relative to the individual expression of *ACTB*, *GAPDH* and *SDHA* as internal control. Normalised data were scaled relative to the expression in control cells at time 0 h, which was defined as one. Bars represent means ± SEM; * = P < 0.05.

There was no significant difference in *IL6* expression in cells co-cultured with *L*. *vaginalis* and *L*. *buchneri* compared with control cells (Fig. 2 A, B). In contrast, *IL6* was significantly more highly expressed in cells co-cultured with *L*. *ruminis*, reaching the highest level (about fourfold) after 4 h in MOI 10 compared with the control (Fig. 2 C). The greatest influence on *IL6* expression was observed in endometrial epithelial cells co-cultured with *L*. *amylovorus*. Expression levels reached a maximum (about 12-fold) after 2 h MOI dependently compared with controls and decreased thereafter, but stayed on a higher level than controls (Fig. 2 D).

### *IL8* mRNA expression in endometrial epithelial cells after co-culturing with different *Lactobacillus* species

**Fig 3:**
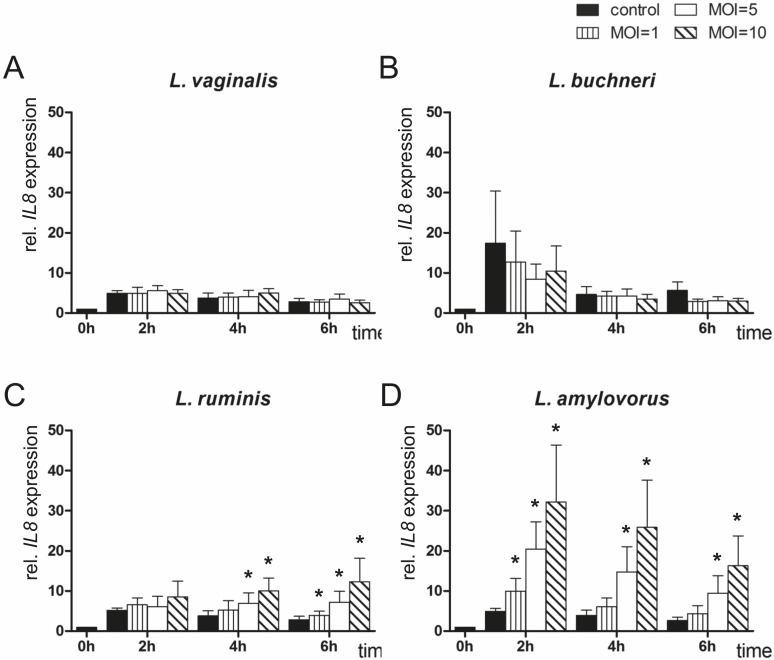
Expression analysis of *IL8* by means of RT-qPCR. *IL8* mRNA expression was determined in bovine endometrial epithelial cells after co-culture with (A) *L*. *vaginalis*; (B) *L*. *buchneri*; (C) *L*. *ruminis*; and (D) *L*. *amylovorus* in MOI 1, 5 and 10 for up to 6 h (n = 5 animals). All expression values were calculated relative to the individual expression of *ACTB*, *GAPDH* and *SDHA* as internal control. Normalised data were scaled relative to the expression in control cells at time 0 h, which was defined as one. Bars represent means ± SEM; * = P < 0.05.

Co-culture with *L*. *vaginalis* and *L*. *buchneri* did not affect *IL8* expression in endometrial epithelial cells compared with untreated cells (Fig. 3 A, B). Increasing *IL8* expression levels were observed after 4 and 6 h in cells co-cultured with *L*. *ruminis* depending on the quantity of MOI (max. fourfold) compared with untreated control cells (Fig. 3 C). *IL8* expression was found on a higher level (two- to sixfold) during the whole time of co-culture of endometrial cells with *L*. *amylovorus* dependently on MOI compared with controls, reaching the highest levels at MOI 10 at each point in time (Fig. 3 D).

### *PTGS2* mRNA expression in endometrial epithelial cells after co-culturing with different *Lactobacillus* species

**Fig 4:**
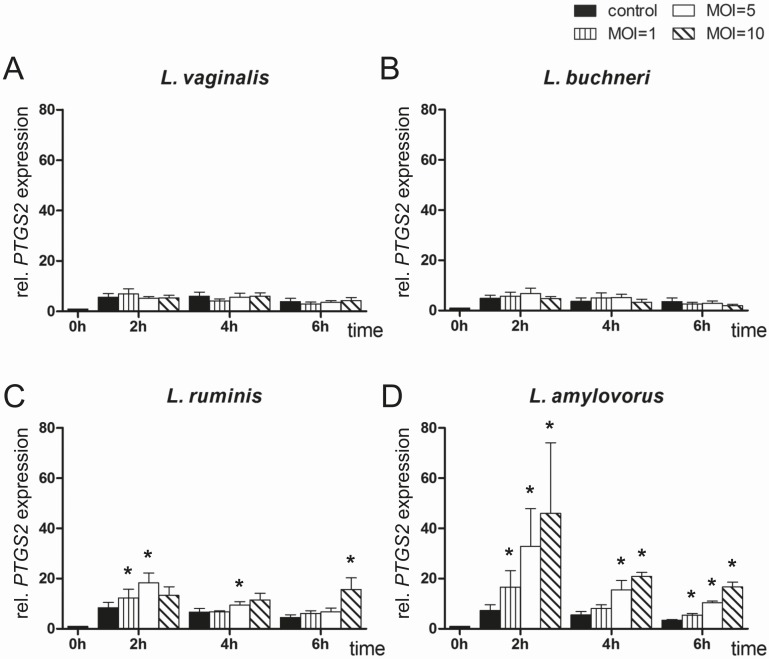
Expression analysis of *PTGS2* by means of RT-qPCR. *PTGS2* mRNA expression was determined in bovine endometrial epithelial cells after co-culture with (A) *L*. *vaginalis*; (B) *L*. *buchneri*; (C) *L*. *ruminis*; and (D) *L*. *amylovorus* in MOI 1, 5 and 10 for up to 6 h (n = 5 animals). All expression values were calculated relative to the individual expression of *ACTB*, *GAPDH* and *SDHA* as internal control. Normalised data were scaled relative to the expression in control cells at time 0 h, which was defined as one. Bars represent means ± SEM; * = P < 0.05.

Comparable to *IL6* and *IL8* expression, *PTGS2* expression was not significantly different in cells co-cultured with *L*. *vaginalis* and *L*. *buchneri* compared with untreated cells (Fig. 4 A, B). A slight increase in *PTGS2* expression (up to ∼fourfold) was detected in endometrial cells co-cultured with *L*. *ruminis* compared with the control (Fig. 4 C). After 2 h exposure, *PTGS2* expression was up to sixfold higher in cells co-cultured with *L*. *amylovorus* and decreased after 4 and 6 h, but remained higher than in control cells in MOI 5 and 10 (Fig. 4 D).

### *TLR2* and *TLR6* mRNA expression in endometrial epithelial cells after co-culturing with different *Lactobacillus* species

*TLR2* and *TLR6* transcripts were present in co-cultured as well as in control cells, but did not differ significantly between these groups (data not shown).

### Release of pro-inflammatory factors by co-cultured endometrial epithelial cells

**Fig 5:**
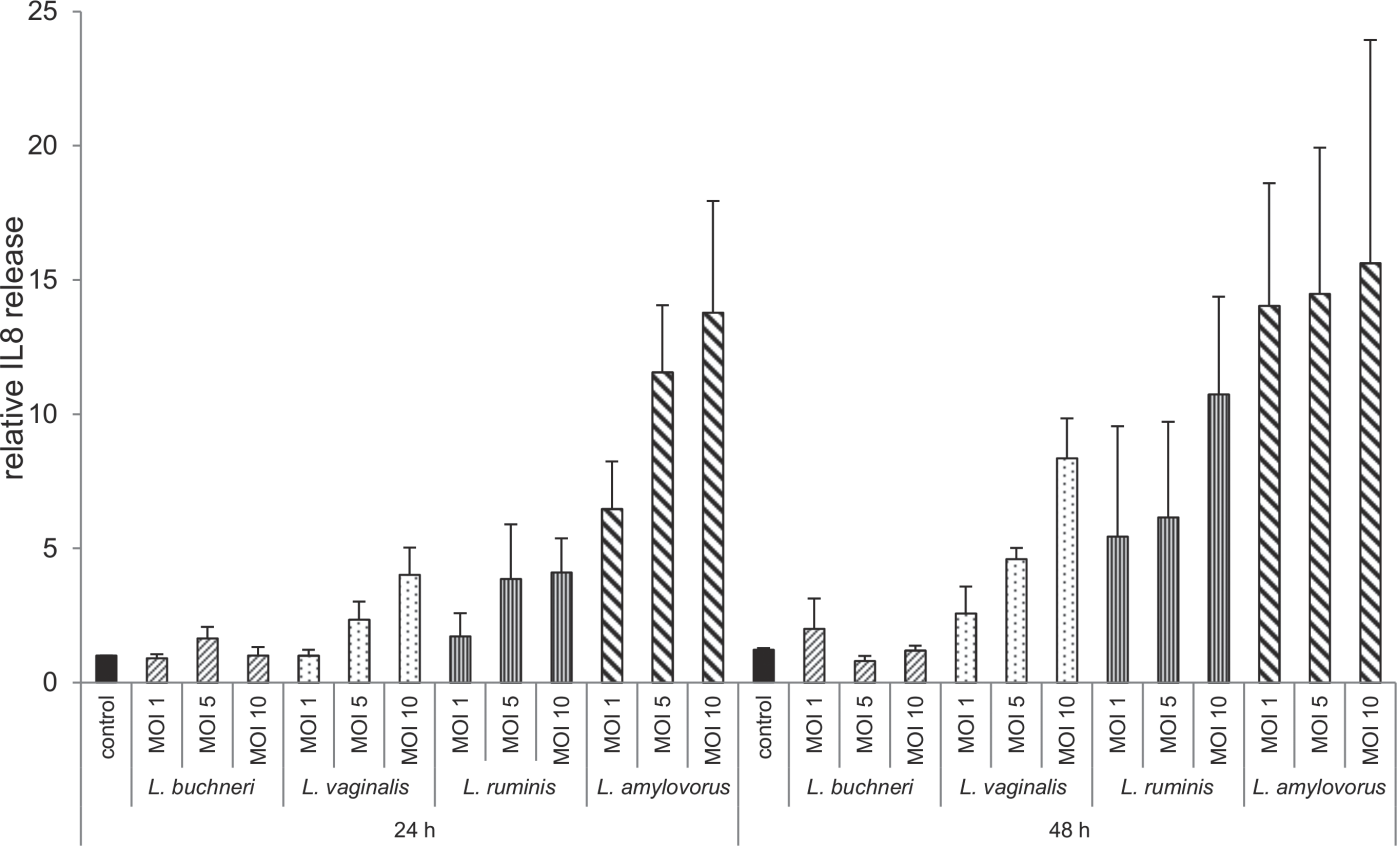
Analysis of IL8 release by means of ELISA. Accumulation of IL8 was measured in supernatants of bovine endometrial epithelial cells co-cultured with *L*. *buchneri*, *L*. *ruminis*, *L*. *amylovorus* and *L*. *vaginalis* in MOI 1, 5 and 10 for 24 and 48 h by ELISA (n = 3 animals). All data were scaled relative to the concentration in supernatants of control cells at time 24 h, which was defined as one. Bars represent means ± SEM.

Concentrations of IL6 remained in supernatants of co-cultured cells as well as in controls under the detection limit of 78 pg/ml of the Bovine IL-6 Screening Set, even after 48 h of co-culture in undiluted samples (data not shown). There was no difference in IL8 release in cells co-cultured with *L*. *buchneri* after 24 and 48 h compared with control cells (Fig. 5). Co-culture of endometrial cells with *L*. *vaginalis* and *L*. *ruminis* caused a similar accumulation of IL8 (up to fourfold) after 24 h compared with the controls. After 48 h, expression levels of IL8 were increased (up to seven- and ninefold in MOI 10) by *L*. *vaginalis* and *L*. *ruminis* compared with the controls, respectively (Fig. 5). The greatest difference in IL8 accumulation compared with untreated cells was detected in endometrial cells co-cultured with *L*. *amylovorus* (Fig. 5). IL8 concentration increased up to 14-fold in MOI 10 after 24 h and remained on this high level (about 12-fold) after 48 h compared with control cells with interindividual variations, respectively. IL8 accumulation differed up to threefold after 24 h and up to 10-fold after 48 h between epithelial cells isolated from different animals in co-culture with *L*. *amylovorus*. Between the epithelial cells obtained from different animals, a high variation was observed in the value range for IL8 (∼ 1–32 pg/ml to 10–150 pg/ml).

A PGE_2_ production by co-cultured as well as untreated epithelial cells could not be measured (detection limit = 15 pg/ml), neither could PGE_2_ be measured in undiluted samples after 48 h of co-culturing (data not shown).

**Fig 6:**
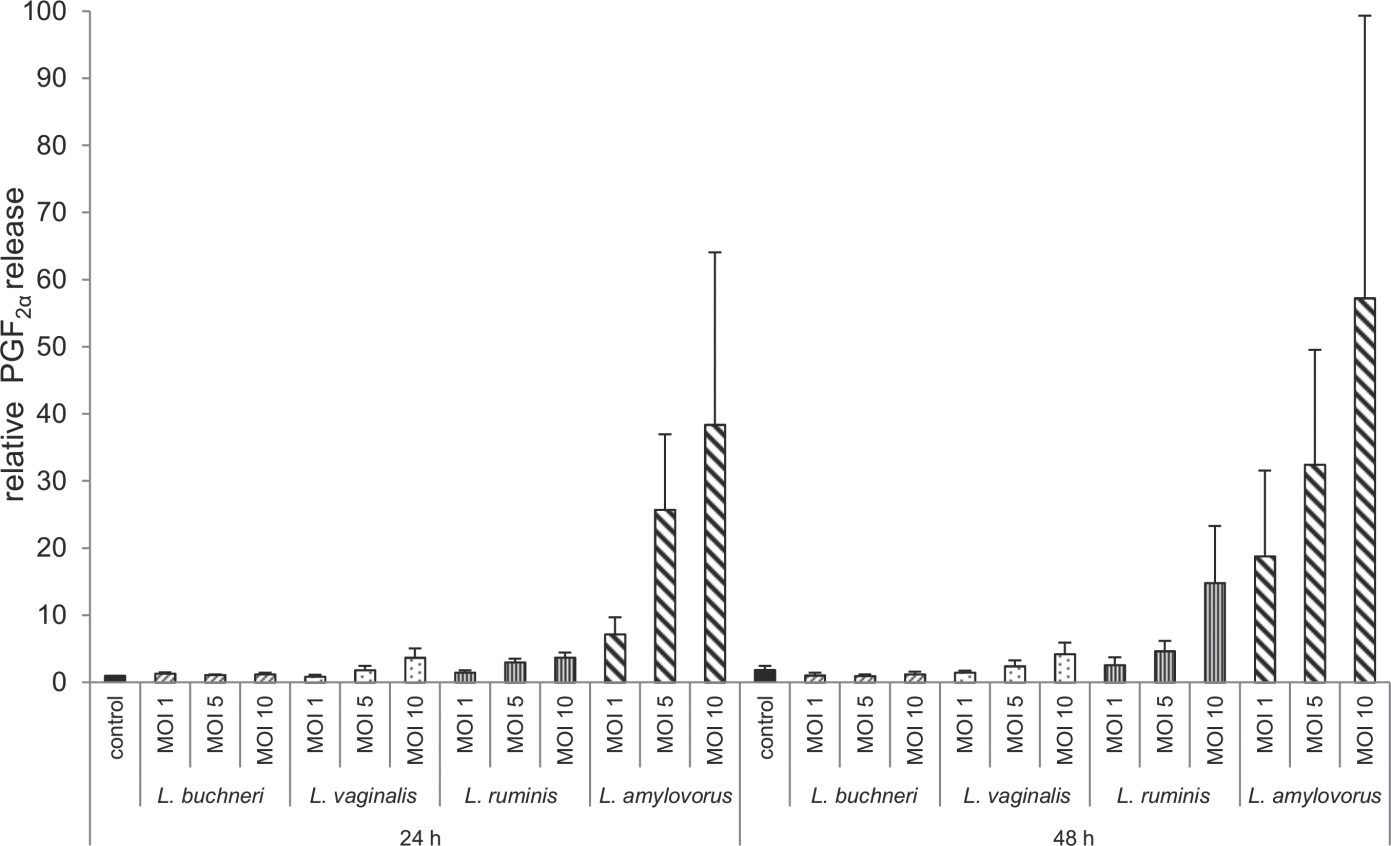
Analysis of PGF_2α_ release by means of EIA. Accumulation of PGF_2α_ was measured in supernatants of bovine endometrial epithelial cells co-cultured with *L*. *buchneri*, *L*. *ruminis*, *L*. *amylovorus* and *L*. *vaginalis* in MOI 1, 5 and 10 for 24 and 48 h by EIA (n = 3 animals). All data were scaled relative to the concentration in supernatants of control cells at time 24 h, which was defined as one. Bars represent means ± SEM.

*L*. *buchneri* did not influence PGF_2α_ accumulation in cell culture supernatants after 24 and 48 h compared with untreated cells (Fig. 6). PGF_2α_ release by endometrial epithelial cells slightly increased in co-culture with *L*. *vaginalis* and *L*. *ruminis* (up to fourfold after 24 h; up to two- and eightfold after 48 h, respectively) compared with control cells (Fig. 6). The highest release of PGF_2α_ by endometrial epithelial cells was detected in co-culture with *L*. *amylovorus* (Fig. 6). The difference was up to 38-fold after 24 h and up to 31-fold after 48 h in comparison between co-cultured and untreated cells with obvious interindividual variations (value range between around 100 pg/ml and 50 ng/ml). PGF_2α_ production in the presence of *L*. *amylovorus* differed up to 20-fold after 24 h and up to 33-fold after 48 h in comparison between endometrial epithelial cells isolated from different animals.

## Discussion

The microbiota present in the uterus of postpartum cows has been the subject of various studies over the last few decades [1,5,39,40], whereby the main focus was on pathogenic bacteria that cause uterine diseases such as metritis, and clinical and subclinical endometritis [2,41,42]. This is the first study describing in depth the isolation and molecular characterisation of commensal lactobacilli from the bovine uterus. Several *Lactobacillus* spp. as well as *Weissella* spp. and *Pediococcus* sp. were isolated from endometrial cytobrush samples. Similarly, *L*. *buchneri* and *L*. *brevis* were present in the bovine vagina [43]. Machado et al. [44] found the genus *Lactobacillus* by metagenomic pyrosequencing of the 16S rRNA gene in 41% of uterine lavage samples from healthy cows. However, the presence of alive lactobacilli was not demonstrated in that study. *In vitro* experiments in the present study were performed with three isolates of *Lactobacillus* spp. in comparison to *L*. *vaginalis*, which is part of the microbiota in the human vagina [45]. This strain was chosen as being representative of the commensal, non-pathogenic bacteria in the female genital tract.

Bacteria that invade the bovine uterus can be categorised into three groups according to their expected pathogenic potential [1]: (1) pathogens that cause uterine endometrial lesions; (2) potential pathogens commonly not associated with endometrial lesions, but frequently isolated from the bovine uterine lumen and cases of endometritis; and (3) opportunist contaminants transiently isolated from the uterine lumen and not associated with endometritis. In the present study, viability assays demonstrated that *L*. *buchneri*, *L*. *ruminis*, *L*. *amylovorus* and *L*. *vaginalis* have no negative influence on the survival and vitality of co-cultured endometrial epithelial cells. These results are comparable with findings of human vaginal epithelial cells exposed to *L*. *rhamnosus* and *L*. *reuteri* [25]. The viability of the co-cultured vaginal cells was not affected by lactobacilli. This indicates that also in the bovine uterus lactobacilli belong to the commensal microbiota not associated with endometrial lesions.

Even though the viability of endometrial cells was not influenced in the present study, the ability of lactic acid bacteria to modulate the host immune response has been described in cattle and humans [10,46,47], where increased cytokine expression is involved [48]. Therefore, one aim of this study was to examine the capability of different *Lactobacillus* species to modulate inflammatory responses in bovine endometrial epithelial cells. Interleukins such as IL1A, IL6 and IL8 play an important role in causing inflammation [15]. The pro-inflammatory cytokine IL1A stimulates the production of PGE_2_, platelet activating factor as well as nitric oxide and promotes the infiltration of inflammatory cells into the extravascular space [49]. In addition, IL6 promotes inflammation by the activation of acute phase response and stimulation of lymphocytes [50]. In cattle, IL6 takes part in the immune defence of the endometrium against bacterial invasion [51]. The chemokine IL8 attracts neutrophils to inflammatory sites and activates them, resulting in the influx of PMNs into the uterine lumen, which is essential for bacterial clearance after calving [14].

In the present study, *L*. *ruminis* and especially *L*. *amylovorus* caused higher mRNA expression of *IL1A*, *IL6* and *IL8* in co-cultured endometrial epithelial cells than in control cells, showing the ability of lactobacilli to stimulate endometrial immune response. This result was confirmed by the detection of a higher accumulation of IL8 in the supernatants of epithelial cells co-cultured with *L*. *ruminis*, and to a greater degree with *L*. *amylovorus*, than without bacteria. Previous studies have indicated immunomodulatory activities of lactic acid bacteria. In cattle, administration of *Lactococcus lactis* in the mammary gland induced enhanced *IL1B* and *IL8* mRNA expression [48]. Furthermore, in humans, stimulation of human vaginal epithelial cells with *L*. *rhamnosus* and *L*. *reuteri* resulted in an increased *IL1A*, *IL1B* and *IL8* mRNA expression [25]. Co-culture with *L*. *fermentum* or *L*. *salivarius* isolated from human breast milk induced enhanced IL8 production in peripheral blood mononuclear cells [26]. In contrast, colonisation of human vaginal epithelial cell multilayer cultures with *L*. *crispatus* or *L*. *jensenii* did not alter IL8 secretion [52]. Our results are consistent with these findings. *IL8* expression and release were not influenced by *L*. *buchneri*, assuming that lactobacilli isolated from bovine uteri have different immunomodulatory properties.

Not only commensal bacteria, but also several pathogenic bacteria have been shown to stimulate the expression and release of interleukins in endometrial cells. Treatment with *E*. *coli* for 6 h increased *IL1B*, *IL6* and *IL8* mRNA expression (17-fold to 121-fold) in bovine endometrial epithelial cells compared with untreated cells [53]. LPS purified from *E*. *coli* stimulated mRNA expression of *IL1B*, *IL6* and *IL8* (tenfold to 110-fold) in endometrial epithelial cells compared with control cells [54]. In addition, IL1B, IL6 and IL8 production by endometrial explants was increased after treatment with *E*. *coli* or *T*. *pyogenes* compared with controls [55]. Cows with uterine diseases, often associated with pathogens such as *E*. *coli* and *T*. *pyogenes*, are known for increased expression of pro-inflammatory factors in the endometrium. Recently, a study was performed with a potentially pathogenic *Bacillus pumilus* isolated from the bovine uterus [56]. mRNA expression of *IL1A*, *IL6* and *IL8* was around two- to threefold higher in bovine endometrial epithelial cells co-cultured with *B*. *pumilus* than with *L*. *amylovorus* in the present study. These findings show that excessive expression of pro-inflammatory factors by the endometrium supports the development of uterine diseases, but an adequate immune response is necessary for bacterial clearance after parturition. Commensal bacteria such as lactobacilli can moderately contribute to stimulating this immune reaction of the endometrium.

Besides interleukins, prostaglandins are involved in inflammatory processes, but they also have different reproductive functions in mammals as regulators of the oestrous cycle, implantation and parturition [57]. In cattle, PGF_2α_ and PGE_2_ are considered to act in a luteolytic and luteotrophic manner, respectively [58]. Synthesis of PGF_2α_ and PGE_2_ from arachidonic acid in the endometrium is regulated by *PTGS2* [59]. Pathogenic bacteria have been shown to influence *PTGS2* expression and prostaglandin production. Stimulation of bovine endometrial cells with *E*. *coli* or LPS increased the *PTGS2* mRNA expression and release of PGF_2α_ and PGE_2_ [16]. Endometrial explants treated with LPS produced an increased ratio of PGE_2_ to PGF_2α_. This switch from PGF_2α_ to PGE_2_ production was confirmed by findings of higher concentrations of PGE_2_ than PGF_2α_ in supernatants of bovine endometrial cells stimulated with LPS [60]. In addition, a bacteria-free filtrate of *T*. *pyogenes* was able to enhance PGF_2α_ and PGE_2_ production in endometrial explants and bovine endometrial cells [61]. The endocrine switch in prostaglandin secretion from PGF_2α_ to PGE_2_ by bovine endometrium has an influence on the fertility of cows assuming a mechanism for prolonged luteal phases in animals with uterine disease [60]. This study showed that *L*. *ruminis* and *L*. *amylovorus* isolated from bovine uteri also induced increased *PTGS2* expression in bovine endometrial epithelial cells. A PGF_2α_ release by endometrial epithelial cells was mainly increased by *L*. *amylovorus* promoting rather a luteolytic condition in the uterine environment. In contrast to the earlier findings mentioned above, we observed no influence of lactobacilli on PGE_2_ production, which remained under the detection limit. However, previous studies support the finding of PGF_2α_ production by epithelial cells and PGE_2_ by stromal cells [16,62].

In the human genital tract, lactobacilli are well studied, recognising their beneficial properties for women’s health [9,12,63]. However, in cattle the role of lactobacilli in the uterus is virtually unstudied. Only one study demonstrated the stimulation of cell defence mechanisms of the bovine endometrium by intrauterine infusion of selected *Lactobacillus* strains [64]. The survival of the infused lactobacilli was confirmed for up to 12 days and cellular infiltration of the endometrium mostly by mononuclear cells was shown. Moreover, an intravaginal administration of different lactic acid bacteria resulted in a reduced incidence of purulent vaginal discharges in dairy cows [65]. Our outcome supports the finding that some *Lactobacillus* species have the ability to act as an immunostimulant to the female genital tract. Immunostimulatory properties of a *L*. *ruminis* strain have been shown in humans [66]. The finding of pro-inflammatory flagellin proteins produced by a *L*. *ruminis* strain could be a further explanation of immunostimulatory properties of *L*. *ruminis* [67].

In conclusion, the results of the present study demonstrate that there are *Lactobacillus* species present in the bovine uterus possessing immunomodulatory properties of endometrial cells. In particular, *L*. *amylovorus* and *L*. *ruminis* increased mRNA expression of the pro-inflammatory factors *IL1A*, *IL6* and *IL8* as well as *PTGS2* in bovine endometrial epithelial cells, which was confirmed by enhanced IL8 and PGF_2α_ concentrations in cell culture supernatants. All examined *Lactobacillus* species had no negative influence on the viability of co-cultured bovine endometrial epithelial cells. These findings support the hypothesis that the presence of lactobacilli may stimulate the immune response without showing cytotoxic effects on the endometrium.

## Supporting Information

S1 Fig Viability evaluation of co-cultured bovine endometrial epithelial cells by means of trypan blue staining. Bovine endometrial epithelial cells were stained with trypan blue after 96 h of co-culture (A) with *L*. *buchneri* in MOI 10; (B) with *L*. *ruminis* in MOI 10; (C) with *L*. *amylovorus* in MOI 10; (D) with *L*. *vaginalis* in MOI 10; (E) control. Dead cells were stained in blue. 100x magnification.
(TIF)

S2 Fig Viability evaluation of co-cultured bovine endometrial epithelial cells by means of ethidium bromide/acridine orange staining. Bovine endometrial epithelial cells were stained with ethidium bromide/acridine orange after 72 h of co-culture (A) with *L*. *buchneri* in MOI 10; (B) with *L*. *ruminis* in MOI 10; (C) with *L*. *amylovorus* in MOI 10; (D) with *L*. *vaginalis* in MOI 10; (E) control. The nuclei of dead cells were stained in red and living cells in green. 200x magnification.
(TIF)
