# Supplementary figures and images for: Detection and Characterisation of Lactobacillus spp. in the Bovine Uterus and Their Influence on Bovine Endometrial Epithelial Cells In Vitro
Source: PLoS One. 2015 Mar 24;10(3):e0119793. doi: 10.1371/journal.pone.0119793 (PMC4372290; doi:10.1371/journal.pone.0119793)

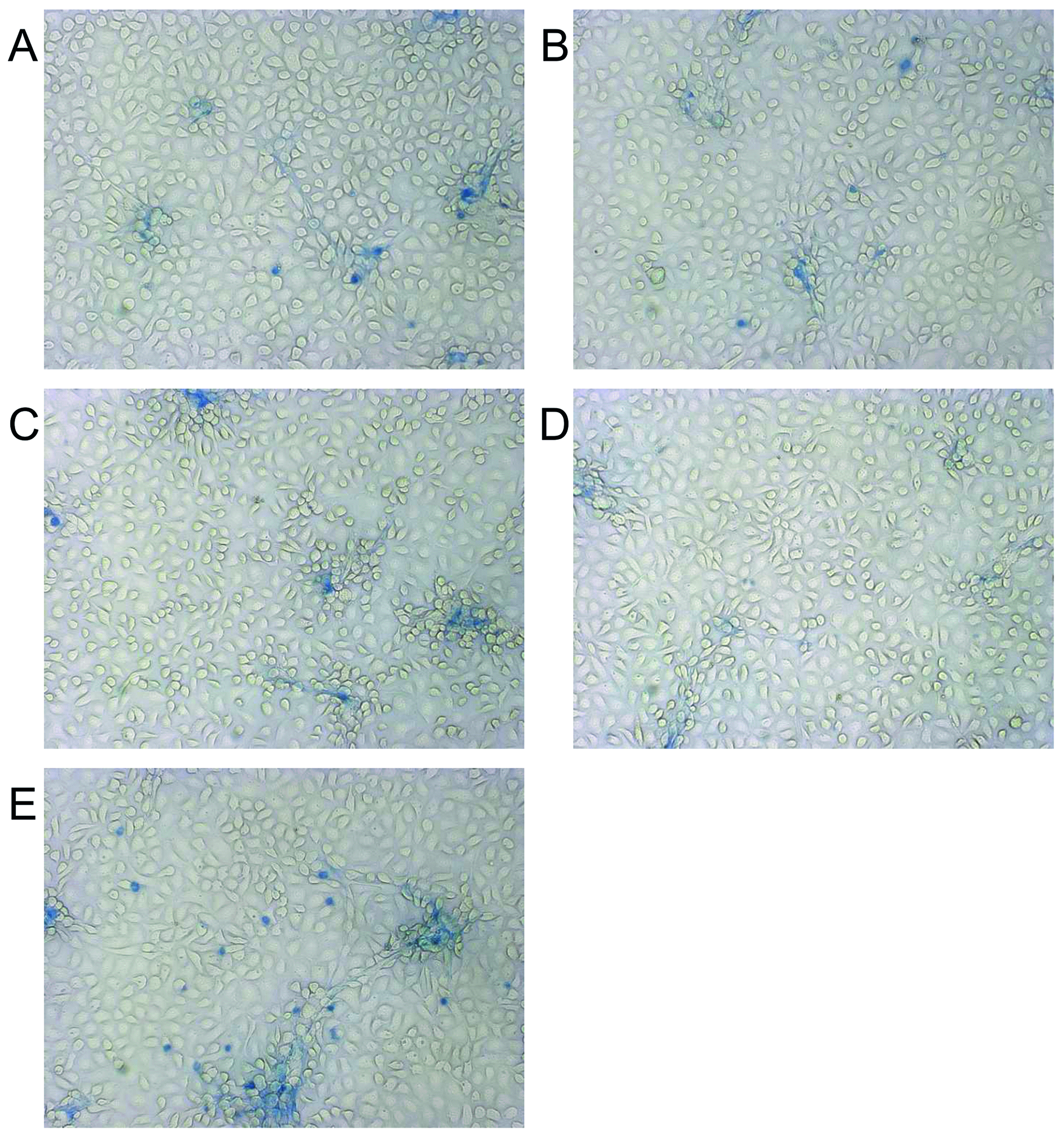

Supplement: S1 Fig — Bovine endometrial epithelial cells were stained with trypan blue after 96 h of co-culture (A) with L. buchneri in MOI 10; (B) with L. ruminis in MOI 10; (C) with L. amylovorus in MOI 10; (D) with L. vaginalis in MOI 10; (E) control. Dead cells were stained in blue. 100x magnification. (TIF) [file pone.0119793.s001.tif]

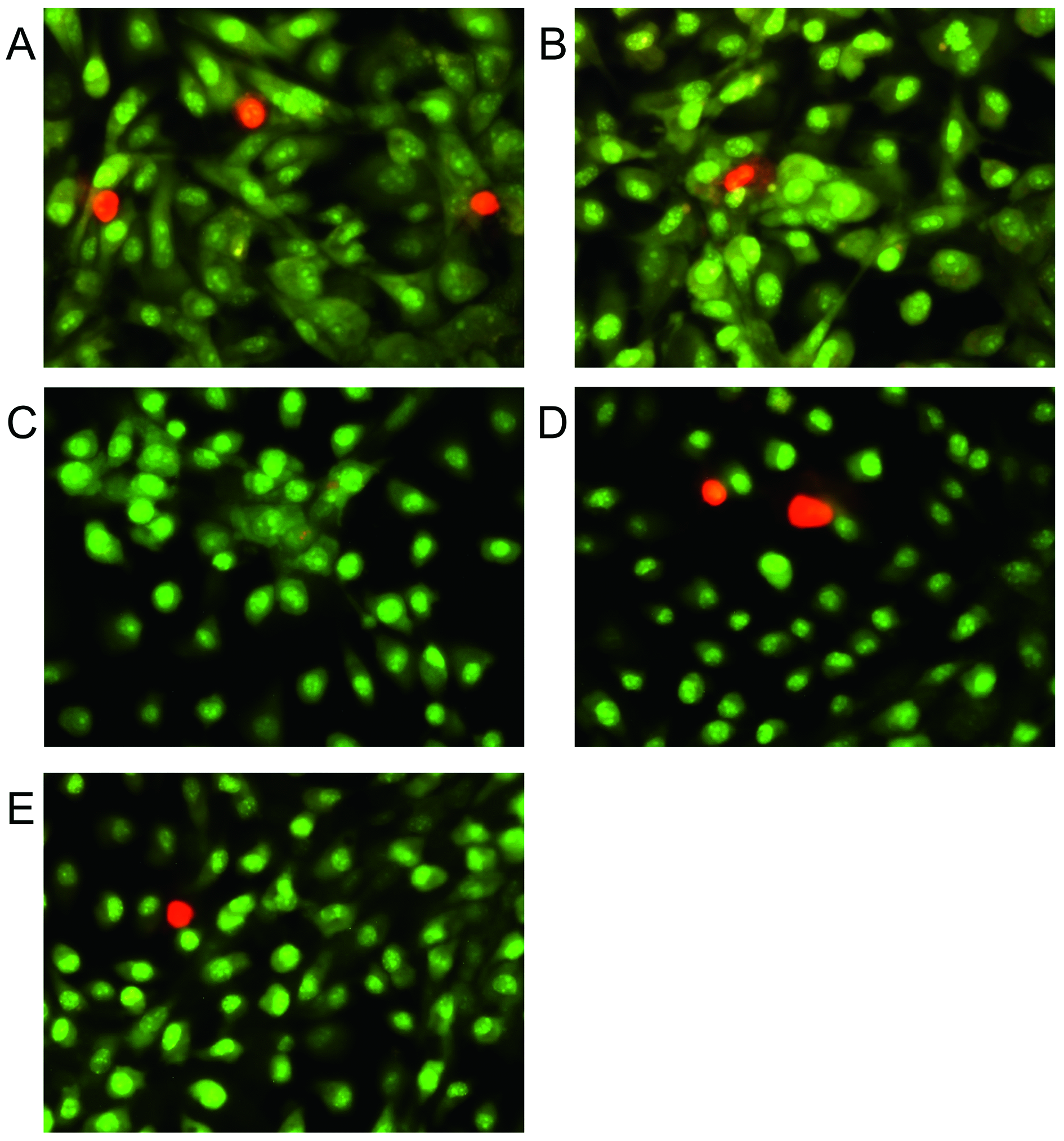

Supplement: S2 Fig — Bovine endometrial epithelial cells were stained with ethidium bromide/acridine orange after 72 h of co-culture (A) with L. buchneri in MOI 10; (B) with L. ruminis in MOI 10; (C) with L. amylovorus in MOI 10; (D) with L. vaginalis in MOI 10; (E) control. The nuclei of dead cells were stained in red and living cells in green. 200x magnification. (TIF) [file pone.0119793.s002.tif]
